# Lifetime Trauma Exposure and Posttraumatic Stress Disorder Among African Americans and Black Caribbeans by Sex and Ethnicity

**DOI:** 10.3389/fpsyt.2022.889060

**Published:** 2022-06-21

**Authors:** Sophia Maria Gran-Ruaz, Robert Joseph Taylor, Grace Jacob, Monnica T. Williams

**Affiliations:** ^1^School of Psychology, University of Ottawa, Ottawa, ON, Canada; ^2^School of Social Work, University of Michigan, Ann Arbor, MI, United States; ^3^School of Cellular and Molecular Medicine, University of Ottawa, Ottawa, ON, Canada

**Keywords:** African American (AA), Black Caribbean American, trauma, sex, posttraumatic stress disorder (PTSD), help-seeking

## Abstract

**Objective:**

Posttraumatic stress disorder (PTSD) is a debilitating disorder requiring timely diagnosis and treatment, with special attention needed for Black populations in the U.S. Yet, stakeholders often fail to recognize Black communities' heterogeneous ethnic composition, thus not allowing diverse sociocultural realities to inform PTSD interventions. This study aims to characterize sex and ethnic differences in lifetime trauma exposure, lifetime PTSD diagnosis and symptoms, and help-seeking among the African Americans and Black Caribbeans in the U.S.

**Method:**

This study relied on data from the National Survey of American Life 2001–2003 (NSAL) to investigate the lifetime exposure to traumatic events and prevalence of a clinical PTSD diagnosis based on the DSM-IV among African American (*n* = 3,570) and Black Caribbean (*n* = 1,623) adults. 44.5% of respondents were men and 55.5% were women. Logistic regression was utilized to investigate the impact of traumatic events on PTSD.

**Results:**

Several ethnic and sex differences in exposure to potentially traumatic events were identified. African American respondents were more likely to experience spousal abuse and toxin exposure than their Black Caribbean counterparts. Black Caribbeans reported higher lifetime exposure to muggings, natural disasters, harsh parental discipline, being a civilian living in terror and/or being a refugee than African American respondents. Specific to sex, Black men reported more events of combat, a peacekeeper/relief worker, being mugged, toxin exposure, seeing atrocities, and/or injuring someone. Black women were more likely to have been rape/sexual assault and/or intimate partner violence victims. The assaultive violence trauma type was most predictive of lifetime PTSD diagnosis among Black Americans. African American women were more likely to report PTSD symptoms than men, with almost no significant differences in Black Caribbean men and women. Approximately half of Black Americans sought help for their worst traumatic event, commonly engaging family/friends, psychiatrists, and mental health professionals. Further, there were almost no ethnic and sex differences related to professional and non-professional help sought.

**Conclusion:**

Future PTSD-related research should aim to characterize the heterogenous experiences of potentially traumatic events within different Black communities. Clinicians working with Black clients should strive to understand the limitations within their tools/interventions in meeting the needs of diverse groups.

## 1. Introduction

When exposed to an event, either directly or indirectly, that causes stress, fear or hopelessness, it is a natural human response to experience a range of negative physical and psychological effects. However, for 6.8% of Americans, these negative effects lead to lasting impacts on one's quality of life and a subsequent diagnosis of posttraumatic stress disorder [PTSD; (1)]. Known physical and mental health consequences of PTSD include autoimmune diseases (2–4), cancer (5, 6), cardiovascular disease (7, 8), chronic pain (9–11), depression (12, 13), eating disorders (14, 15), generalized anxiety disorder (16, 17), insomnia (18, 19), obsessive-compulsive disorder (20, 21), substance abuse (22–24), and suicide (22, 25). In addition, research shows that PTSD impairs one's social wellbeing by increasing marital problems, family conflict, and occupational instability (26). In examining PTSD within American veteran sufferers, the economic burden was estimated to gross $8.3 billion (27). Hence, why it is essential individuals with PTSD are diagnosed and receive timely and appropriate treatment.

In consideration of PTSD and ethnoracial identity, a review by Asnaani and Hall-Clark (28) notes limited and conflicting evidence on the existence of racial differences in PTSD development. However, for those noting a significant effect, Black Americans exhibited the highest prevalence rates compared to other explored racial groups (29–31). Himle and colleagues (32) estimated that the lifetime prevalence of PTSD within Black Americans was 9.1%. However, within the current climate of racial injustice and coronavirus pandemic, it could be higher today. Others have gone further to suggest Black American PTSD sufferers experience more severe symptoms than other racial groups in terms of the number of symptoms endorsed and related distress [e.g., (33–36)]. In trying to explain the aforementioned gaps in prevalence and symptom severity, several researchers cite greater disparities within housing and neighbourhood quality (37, 38), experiences of racism and discrimination (39, 40), intergenerational trauma (41, 42), income (43), educational achievement (31), etc., amongst Black Americans compared to other groups. Others suggest that the differences in prevalence and symptom severity seen in Black Americans are a function of their being more vulnerable to select potentially traumatic events compared to other racial groups. Moreover, Roberts and team (31) note, only 35.5% of Black American PTSD sufferers sought treatment, compared to 53.3% of White sufferers.

As past studies have shown, Black people in the United States have the highest lifetime prevalence of PTSD (29–31). However, Black Americans are a very heterogeneous group, with potentially diverse sociocultural contexts. As of 2019, there are an estimated 46.7 million Black Americans living in the United States, making up 14% of the national population (44). Some 87% of Black Americans identify themselves as solely “Black,” 8% as multi-racial (i.e., Black and another race), and 5% as “Black-Hispanic” (44). Linguistically, English is most predominantly spoken (96%), but Spanish (3%), French or Haitian Creole (2%) and/or Amharic and other Ethiopian languages are not uncommon (1%) (45). Also of note, 10% of the Black American population are foreign-born and thus immigrated to the United States (45). Most Black immigrants originated from either the Caribbean (46%) or Africa (42%), with the top five birthplaces being Jamaica, Haiti, Nigeria, Ethiopia, and the Dominican Republic (46). Considering year of immigration, 42% of Black Caribbean immigrants arrived before 1990 (46). By contrast only 13% of African immigrants arrived before 1990, with most arriving in the year 2000 or later (46). Further, 9% of US-born Black Americans have at least one foreign-born parent (47).

A failure to understand and recognize ethnoracial heterogeneity can prove a harmful oversight in ensuring underserved groups have access to the culturally relevant interventions they need to safely and efficaciously treat their PTSD. To this point, some ground has been made: experts acknowledge that the vast amount of research and research-informed outputs generated to date were created with/for majority White populations (48). Many are also aware that what has been found to characterize White people's psychological experience, does not always overlap neatly onto the minority experience (49). However, as the field of psychology moves towards building an understanding of the unique experiences of non-White populations, they must take it one step further, respecting that there are still many layers of within-group diversity not yet explored. More specifically, aggregating all Black Americans into one ethnoracial category instead of the highly diverse groups they are, prevents mental health providers from understanding how different psychosocial contexts influence said groups (49, 50) and their treatment. For those few researchers who have explored ethnoracial differences in other common mental health disorders within Black American subgroups, within-group differences in prevalence and presentation have been noted [e.g., major depressive disorder (51), substance use disorder (52), obsessive compulsive disorder (53), anxiety disorders (32), etc]. As such, one can not help but wonder: in what ways do Black American subgroups, like the large African American and Black Caribbean subgroups, differ in their exposure to potentially traumatic events and their PTSD presentations. How might differences in history, customs, knowledge, beliefs, values, languages, etc. impact the risk of PTSD development, PTSD presentation, help-seeking behaviours, and ultimately treatment?

Similarly, the experiences of men and women in terms of potential traumatic event-type exposure, PTSD development, and resultant treatment outcomes can be very different between the sexes. Absent consideration of ethnicity, a landmark meta-analysis by Tolin and Foa considering sex-specific risk to traumatic events and PTSD, found men were more likely than women to report having experienced a potentially traumatic life-event (54). Nevertheless, the authors of said review also noted women were more likely than their male counterparts to meet diagnostic criteria for PTSD and to report a greater severity of PTSD-related symptoms. Such findings beg the question: do men and women differ in the types of traumatic events they experience in their lives, and do certain events lead to a greater vulnerability in PTSD development? While Tolin and Foa go on to suggest there are other factors at play beyond strictly the potentially traumatic event-type in explaining the PTSD sex differences seen, little has been done at even the most basic level to characterize the unique experiences of trauma exposure in Black American men and women.

For said reasons, this study aims to consolidate and supplement the existing literature on sex and ethnoracial differences within Black Americans related to traumatic event exposure over the lifespan, as well as the lifetime diagnosis of PTSD, its symptomatology, and related help-seeking behaviors. To do so, the article will first present related findings from the *National Survey of American Life* (NSAL) 2001–2003, regarding men and women of African (“African Americans”) and Caribbean decent (“Black Caribbeans”). While almost 20 years old, NSAL remains the most comprehensive and detailed study of Black American mental health ever completed and one of the only that allows for such ethnic subgroup analysis. Once presented, the article will situate these findings within the broader body of research and present actionable recommendations for researchers and clinicians looking to better serve these diverse communities.

## 2. Method

### 2.1. Data Source, Design, and Sample

NSAL is one of the *Collaborative Psychiatric Epidemiology Surveys* (CPES). The primary objective of the CPES initiative was to collect data regarding the prevalence of mental disorders, impairments associated with these disorders, and their treatment patterns from representative samples of Americans. Diagnostic interviews were undertaken between February 2001 and March 2003 by trained interviewers from the participants' local communities, with race/ethnic matching employed to optimize the interview experience. Moreover, NSAL focuses on *Diagnostic and Statistical Manual of Mental Disorders* (DSM-IV) disorders, psychological distress, and service use among African American and Black Caribbean adults. At the time of its collection, the NSAL was considered the “most comprehensive and detailed study of mental health of Americans of African descent ever completed” and this remains true today [(55), p. 196]. The final NSAL adult national sample, representative of the Black population in the United States at the time, consisted of *n* = 3,570 African Americans, *n* = 1,623 Black Caribbeans, and *n* = 891 non-Hispanic Whites for a total of 6,082 participants over 18 years of age. 44.0% of African American respondents were men and 56.0% were women, with a mean age of 42.3 (SE = 0.52). 50.9% of Black Caribbean respondents were men and 49.1% were women, with a mean age of 40.3 (SE = 0.84). The study design and sampling method of the NSAL have been documented in great detail (56–58). The NSAL data collection was approved by the University of Michigan's Institutional Review Board.

### 2.2. Measures

#### Potential Traumatic Events

Respondents in the NSAL were asked whether they had experienced occurrences from a list of 27 potential traumatic events. They were also asked two open-ended questions that inquired about any other event not on the list or any “private” event the respondent did not want to disclose. These potential traumatic events were further classified into six groups: any war-related trauma, any assaultive violence, any other injury or shocking event, trauma experienced by close friend/relatives, respondent caused trauma, and other non-specified trauma. The full list of potential traumatic events is in Table 1. Of note, the potential traumatic event categories reported on are consistent with previous research in the traumatic stress field [e.g., (31)].

**Table 1:** Sex and ethnic differences in lifetime exposure to potentially traumatic events for African Americans and Black Caribbeans.

**Category**	**African Americans**			**Black Caribbeans**		
**Event Type**	**Men**	**Women**	**Total**	**Men**	**Women**	**Total**
	**%(*N*)**	**%(*N*)**	**%(*N*)**	**%(*N*)**	**%(*N*)**	**%(*N*)**
**Any trauma** ^‡^	82.28 (1,035)	79.16 (1,811)	80.53 (2,846)	85.98 (525)	78.21 (769)	82.16 (1,294)
**Any war-related trauma**^†^^†^^†^^‡^^‡^^‡^	17.02 (224)	1.63 (30)	8.40 (254)	13.96 (85)	3.98 (34)	9.06 (119)
Combat^†^^†^^†^^‡^^‡^^‡^	14.28 (184)	0.67 (13)	6.66 (197)	9.05 (44)	0.45 (3)	4.80 (47)
Peacekeeper/relief worker^†^^†^^†^^‡^^‡^	4.06 (54)	0.37 (6)	2.00 (60)	2.75 (17)	0.39 (7)	1.58 (24)
Unarmed civilian in war zone^†^^†^^†^	2.96 (36)	0.67 (12)	1.68 (48)	2.17 (24)	1.42 (17)	1.80 (41)
Refugee*^†^	1.52 (15)	0.55 (9)	0.98 (24)	3.18 (17)	2.13 (14)	2.66 (31)
**Any assaultive violence**	43.66 (530)	47.24 (1,062)	45.66 (1,592)	52.21 (286)	45.21 (449)	48.77 (735)
Raped^†^^†^^†^^‡^^‡^^‡^	1.78 (25)	17.55 (368)	10.60 (393)	2.31 (13)	16.86 (126)	9.48 (139)
Sexually assaulted^†^^†^^†^^‡^	5.25 (63)	19.63 (415)	13.28 (478)	9.49 (32)	18.92(157)	14.14 (189)
Badly beaten by spouse**^†^^†^^†^^‡^^‡^^‡^	1.33 (19)	17.90 (392)	10.60 (411)	0.85 (10)	11.96 (113)	6.34 (123)
Badly beaten by parents*^‡^	5.22 (63)	5.58 (120)	5.42 (183)	12.34 (54)	5.60 (72)	9.01 (126)
Badly beaten by other^†^^†^^†^^‡^^‡^^‡^	9.72 (115)	3.12 (62)	6.02 (177)	11.68 (49)	2.82 (35)	7.30 (84)
Kidnapped ^‡^^‡^^‡^	1.30 (14)	1.84 (43)	1.60 (57)	0.39 (4)	2.48 (18)	1.42 (22)
Stalked^†^^†^^†^	9.27 (120)	16.74 (362)	13.46 (482)	8.90 (52)	16.36 (125)	12.58 (177)
Mugged*^†^^†^^†^^‡^^‡^	36.85 (416)	16.70 (344)	25.57 (760)	42.28(227)	21.30 (214)	31.91 (441)
**Any other injury or shocking event**^†^^†^^†^^‡^	68.14 (861)	54.28 (1,236)	60.38 (2,097)	71.58(439)	57.59 (557)	64.71 (996)
Life-threatening car accident^†^^‡^^‡^^‡^	20.31 (253)	16.99 (363)	18.45 (616)	24.63(123)	13.61 (121)	19.18 (244)
Other life-threatening accident^†^^†^^†^^‡^^‡^^‡^	10.52 (133)	3.48 (81)	6.58 (214)	10.58(50)	2.67 (33)	6.67 (83)
Ever life-threatening illness^†^	11.19 (149)	13.93 (285)	12.72 (434)	11.36(57)	10.98 (83)	11.18 (140)
Ever natural disaster***^†^^†^^†^	18.74 (229)	11.11 (249)	14.47 (478)	26.83(146)	20.03 (201)	23.47 (347)
Ever man-made disaster^†^	8.33 (98)	5.52 (116)	6.76 (214)	10.39(28)	6.40 (58)	8.42 (86)
Ever exposed to toxins*^†^^†^^†^^‡^	10.82 (129)	3.04 (60)	6.45 (189)	5.25(39)	2.04 (22)	3.66 (61)
Ever live as civilian among terror***^†^^†^^†^^‡^^‡^	4.37 (46)	1.61 (27)	2.82 (73)	12.69(51)	4.42 (38)	8.60 (89)
Ever see someone badly injured^†^^†^^†^	46.00 (543)	23.67 (509)	33.50 (1,052)	45.82 (262)	30.20 (268)	38.11 (530)
Ever see physical fights^†^^†^^†^	16.01 (193)	21.32 (461)	18.98 (654)	14.85 (79)	14.69 (149)	14.77 (228)
Ever see atrocities^†^^†^^†^^‡^^‡^	10.03 (119)	1.78 (40)	5.41 (159)	12.25 (60)	2.33 (13)	7.35 (73)
**Trauma experienced by close friend/relatives**	52.37 (650)	56.02 (1,256)	54.41 (1,906)	51.58 (277)	48.60 (437)	50.12 (714)
Ever had child w/ life-threatening illness^†^^†^	8.72 (107)	12.01 (261)	10.56 (368)	5.28 (22)	8.51 (64)	6.87 (86)
Ever anyone close die unexpectedly	49.10 (581)	50.05 (1,084)	49.63 (1665)	48.52 (256)	43.94 (384)	46.26 (640)
Ever anyone close had trauma	9.42 (104)	10.67 (222)	10.12 (326)	8.53 (50)	9.05 (82)	8.78 (132)
**Respondent Caused Trauma**^†^^†^^†^^‡^^‡^^‡^	6.94 (87)	2.78 (62)	4.61 (149)	9.70 (37)	1.88 (13)	5.86 (50)
Ever accidentally injure other^†^^†^^‡^^‡^	2.62 (34)	0.99 (24)	1.70 (58)	4.56 (19)	0.56 (5)	2.58 (24)
Ever purposely injure other^†^^†^^†^^‡^^‡^	5.81 (69)	2.07 (44)	3.71 (113)	8.36 (25)	1.56 (10)	5.00 (35)
Other non-specified trauma**	10.55 (128)	12.11 (265)	11.43 (393)	17.99 (79)	14.18 (152)	16.12 (231)
Ever experience other trauma^‡^^‡^	5.36 (61)	3.56 (80)	4.35 (141)	8.37 (28)	3.95 (49)	6.18 (77)
Ever trauma not want to report**	7.10 (82)	9.67 (199)	8.54 (281)	14.28 (59)	11.35 (111)	12.83 (170)

*p < 0.05; **p < 0.01; ***p < 0.001 for significant ethnicity differences.

*†p < 0.05; ††p < 0.01; †††p < 0.001 for significant sex differences among African Americans*.

*‡p < 0.05; ‡‡p < 0.01; ‡‡‡p < 0.001 for significant sex differences among Black Caribbeans*.

#### PTSD

Lifetime posttraumatic stress disorder which was assessed using the DSM-IV *World Mental Health Composite International Diagnostic Interview* (WMH-CIDI), a fully structured diagnostic interview (employs a binary scale). Validation studies have found that CIDI diagnoses of PTSD are concordant with independent clinical diagnoses of PTSD (59).

#### PTSD Symptoms

Twelve symptoms of PTSD are examined. These include: purposely staying away from places, people; loss of interest in doing things; having trouble feeling normal feelings like love, happiness; and having repeated unpleasant dreams about the event.

#### Treatment

Respondents who had a traumatic experience are asked about whether they talked to a medical doctor or other professional about their reactions to their worst traumatic event. Professionals included psychiatrists, psychologists, counselors, spiritual advisors, herbalists, acupuncturists, and other healing professionals. Those who did not seek professional help were asked about the reasons that they did not seek it.

### 2.3. Data Analysis

Percentages represent weighted proportions based on the distribution of African Americans and Black Caribbeans in the population. Bivariate associations are tested with the Rao-Scott Chi Square which is a complex design-corrected measure of association. For the multivariate analyses logistic regression was used. Odds ratio estimates and 95% confidence intervals are presented. The regression coefficients and standard errors take into account the complex multistage clustered design of the NSAL sample, unequal probabilities of selection, non-response, and poststratification. All analyses were conducted using SAS 9.13 which uses the Taylor expansion approximation technique for calculating the complex design-based estimates of variance.

## 3. Results

### 3.1. Black Americans and Exposure to Potentially Traumatic Events

#### Common Traumatic Categories and Events Experienced by Black Americans

Table 1 represents the prevalence of lifetime exposure to potential traumatic events for African Americans and Black Caribbeans, by sex, category, and event type. The majority of African Americans (60.4%) and Black Caribbeans (64.7%) reported traumatic experiences involving an *injury or shocking event*, most commonly seeing someone badly injured (33.5% and 38.1%), witnessing a physical fight (19.0% and 14.8%), or being involved in a life-threatening car accident (18.5% and 19.2%). Just over half of African American and Black Caribbean respondents also reported *trauma experienced by a close friend or relative*, most frequently, the unexpected death of someone close to them. Finally, just under half of African American and Black Caribbean respondents reported having experienced a form of *assaultive violence*, most often a mugging.

#### Ethnic Differences in Exposure to Potentially Traumatic Events

As seen in Table 1, African American and Black Caribbean respondents reported similar overall potentially traumatic event exposure prevalence rates of 80.5 and 82.2%. Nevertheless, there were some statistically significant event differences in the type of traumatic event. African Americans were significantly more likely than Black Caribbeans to report having been badly beaten by a spouse, or exposed to toxins (differences of 4.3, and 2.8%, respectively). Conversely, Black Caribbeans were significantly more likely to report having been mugged, having survived a natural disaster, being badly beaten by one's parents, being a civilian living among terror, being a refugee, or having experienced something traumatic they wish not to disclose (differences of 1.7–9.0%).

Also of note, the 9/11 terrorist attack took place during the field period of the NSAL. Given this and Black Caribbeans' high geographical concentration in the Northeast, the authors briefly examined potential influences of this event on the current article's traumatic event analysis (see Table 2). The analysis indicated that there were few pre and post 9/11 differences in traumatic events. Of the 70 relationships there were only 9 significant differences. Almost all these differences were among Black Caribbeans and most importantly only being a refugee was higher post 9/11. The 8 other significant differences found in Table 2. indicated higher trauma pre 9/11 vs. post 9/11. These findings indicate that 9/11 did not have an impact on our traumatic event findings.

**Table 2:** Lifetime exposure to potentially traumatic events for African Americans and Black Caribbeans before and after 9/11/2001.

	**African Americans**		**Black Caribbeans**	
**Category**	**Pre 9/11/2001**	**Post 9/11/2001**	**Pre 9/11/2001**	**Post 9/11/2001**
**Event type**	**%(*N*)**	**%(*N*)**	**%(*N*)**	**%(*N*)**
**Any trauma**	82.06 (1,299)	79.44 (1,547)	84.81 (403)	80.76 (891)
**Any war-related trauma**	9.43 (126)	7.67 (128)	11.60 (45)	7.72 (74)
Combat^∧^^∧^^∧^	7.59 (101)	5.97 (96)	9.97 (22)	2.11 (25)
Peacekeeper/relief worker	1.82 (28)	2.12 (32)	2.21 (8)	1.26 (16)
Unarmed civilian in war zone	2.12 (24)	1.35 (24)	1.32 (11)	2.05 (30)
Refugee^∧^^∧^	1.56 (15)	0.55 (9)	0.84 (14)	3.61 (17)
**Any assaultive violence**	46.28 (713)	45.22 (879)	48.83 (222)	48.73 (513)
Raped	11.84 (194)	9.68 (199)	10.03 (49)	9.20 (90)
Sexually assaulted	13.42 (223)	13.18 (255)	15.75 (65)	13.31 (124)
Badly beaten by spouse^∧^	11.05 (196)	10.27 (215)	8.87 (42)	5.02 (81)
Badly beaten by parents	6.61 (102)	4.55 (81)	9.43 (47)	8.79 (79)
Badly Beaten by Other	6.09 (76)	5.98 (101)	7.02 (25)	7.44 (59)
Kidnapped	1.98 (32)	1.32 (25)	2.38 (9)	0.92 (13)
Stalked	13.00 (215)	13.80 (267)	14.98 (59)	11.33 (118)
Mugged	24.23 (322)	26.55 (438)	32.53 (128)	31.59 (313)
**Any other injury or shocking event** ^∧^^∧^	62.40 (967)	58.93 (1,130)	74.93 (334)	59.32 (662)
Life-threatening car accident	17.60 (265)	19.08 (351)	23.09 (75)	17.15 (169)
Other life-threatening accident	6.20 (97)	6.86 (117)	7.37 (26)	6.31 (57)
Ever life-threatening illness^∧^^∧^	12.76 (200)	12.69 (234)	17.93 (53)	7.68 (87)
Ever natural disaster^∧^^∧^^∧^	14.51 (215)	14.44 (263)	31.80 (139)	19.13 (208)
Ever man-made disaster	6.87 (101)	6.67 (113)	8.28 (28)	8.49 (58)
Ever exposed to toxins	7.25 (97)	5.86 (92)	4.76 (24)	3.09 (37)
Ever live as civilian among terror	2.58 (27)	3.00 (46)	13.21 (29)	6.21 (60)
Ever see someone badly injured	35.60 (513)	31.96 (539)	41.77 (178)	36.20 (352)
Ever see physical fights*_*_	20.97 (311)	17.51 (343)	14.40 (74)	14.97 (154)
Ever see atrocities	5.77 (76)	5.13 (83)	7.69 (25)	7.17 (48)
**Trauma experienced by close friend/relatives** ^∧^	53.66 (863)	54.96 (1,043)	57.92 (218)	46.00 (496)
Ever had kid w/ life-threatening illness^∧^	11.20 (180)	10.09 (188)	9.22 (26)	5.65 (60)
Ever anyone close die unexpectedly	48.53 (757)	50.44 (908)	51.78 (200)	43.39 (440)
Ever anyone close had trauma^∧^	9.92 (136)	10.26 (190)	12.33 (46)	6.94 (86)
**Respondent caused trauma**	4.58 (70)	4.63 (79)	5.69 (18)	5.95 (32)
Ever accidentally injure other	1.72 (27)	1.69 (31)	2.99 (7)	2.37 (17)
Ever purposely injure other	3.64 (51)	3.76 (62)	4.46 (13)	5.28 (22)
**Other non-specified trauma**	11.99 (187)	11.02 (206)	17.39 (83)	15.45 (148)
Ever experience other trauma	3.80 (59)	4.76 (82)	8.13 (23)	5.17 (54)
Ever trauma not want to report	9.65 (142)	7.72 (139)	16.06 (67)	11.15 (103)

*_*_p < 0.05; *_*_*_*_p < 0.01; *_*_*_*_*_*_p < 0.001 for significant time differences among African Americans.

*^∧^p < 0.05; ^∧^^∧^p < 0.01; ^∧^^∧^^∧^p < 0.001 for significant time difference among Black Caribbeans*.

#### Sex Differences in Exposure to Potentially Traumatic Event Types

Table 1 also includes sex-specific differences of lifetime exposure to potentially traumatic events for Black respondents. African American men and women showed similar rates of overall trauma exposure. This finding is dissimilar from Black Caribbeans who indicated men were more likely than women to report *any trauma*. Further, both African American and Black Caribbean men were more likely than women to experience *any war-related trauma, any other injury or shocking event*, and *respondent caused trauma* categories.

Both African American and Black Caribbean men respondents were more likely than women to indicate past event type experiences of combat, being a peacekeeper or relief worker, being mugged, being badly beaten by another, being in a life-threatening car accident or some other life-threatening accident, being exposed to toxins, living as a civilian among terror, seeing atrocities, and ever accidentally or purposely injuring another. In comparison to African American women, African American men were more likely to report being an unarmed civilian in a war zone, being a refugee, having a life-threatening illness, living through a natural or man-made disaster, seeing someone badly injured, and seeing a physical fight. Similarly, Black Caribbean men were more likely to report being badly beaten by parents and experiencing a trauma type not available in the list provided (i.e., “other”) than their female counterparts.

Looking at significantly large sex differences where women reported higher rates of lifetime exposure to potentially traumatic events, both African American and Black Caribbean women respondents indicated an increased likelihood of rape, sexual assault, and physical assault by a spouse. However, African American women were more likely than African American men to report being stalked, and having a child present with a life-threatening illness. Conversely, Black Caribbean women were more likely than African American women to report being kidnapped.

### 3.2. Black Americans and PTSD

#### Potentially Traumatic Event Exposure in Black Americans and Lifetime PTSD Diagnosis

Within the sample, 9.1% of African American participants (5.07% men and 12.27% women) and 8.42% of Black Caribbean participants (8.38% men and 8.46% women) suffered from lifetime PTSD. Table 3 examines the association between lifetime exposure to potentially traumatic events and the lifetime diagnosis of PTSD in Black Americans, by sex, ethnicity, age, and trauma event type. Analyses suggest traumatic experiences of the *assaultive violence* category–specifically rape, being badly beaten by a spouse or by parents, and being stalked–significantly predict the lifetime diagnosis of PTSD in Black Americans. Experiences of a life-threatening illness as well as being a refugee also predicted lifetime PTSD diagnosis.

**Table 3:** Logistic regression models of post-traumatic stress disorder among Black Americans by ethnicity, sex, age and trauma exposure type.

	**Model 1**	**Model 2**
	**(*n* = 4,998)**	**(*n* = 4,903)**
	***OR* (95%CI)**	***OR* (95%CI)**
**Ethnicity**		
African American	1.00	1.00
Black Caribbean	0.95 (0.53–1.61)	0.93 (0.47–1.84)
**Sex**		
Female	2.46 (1.84–3.30)***	1.82 (1.23–2.68)**
Male	1.00	1.00
**Age**		
18–34	1.00	1.00
35–54	0.87 (0.63–1.20)	0.79 (0.56–1.13)
55 and older	0.52 (0.36–0.76)**	0.59 (0.38–0.92)
**Any war-related trauma**		
Combat	–	1.59 (0.81–3.15)
Peacekeeper/relief worker	–	1.40 (0.41–4.74)
Unarmed civilian in war zone	–	0.84 (0.37–1.89)
Refugee	–	3.78 (1.28–11.21)*
**Any assaultive violence**		
Raped	–	2.87 (1.82–4.52)***
Sexually assaulted	–	1.39 (0.85–2.27)
Badly beaten by spouse	–	1.53 (1.13–2.06)**
Badly beaten by parents	–	1.84 (1.04–3.26)*
Badly Beaten by Other	–	1.22 (0.67–2.21)
Kidnapped	–	1.76 (0.87–3.57)
Stalked	–	1.78 (1.20–2.64)**
Mugged	–	0.92 (0.64–1.32)
**Any other injury or**		
**shocking event**		
Life-threatening car accident	–	1.26 (0.86–1.85)
Other life-threatening accident	–	1.61 (0.94–2.74)
Ever life-threatening illness	–	2.07 (1.34–3.21)**
Ever natural disaster	–	0.85 (0.53–1.36)
Ever man-made disaster	–	1.05 (0.54–2.06)
Ever exposed to toxins	–	1.23 (0.62–2.43)
Ever live as civilian among terror	–	0.73 (0.31–1.73)
Ever see someone badly injured	–	1.15 (0.82–1.62)
Ever see physical fights	–	1.33 (0.94–1.90)
Ever see atrocities	–	0.79 (0.39–1.62)
**Trauma experienced by close**		
**friend/relatives**		
Ever had kid w/ life-threatening illness	–	1.29 (0.81–2.05)
Ever anyone close die unexpectedly	–	1.29 (0.97–1.72)
Ever anyone close had trauma	–	1.17 (0.76–1.80)
**Respondent caused trauma**		
Ever accidentally injure other	–	0.60 (0.15–2.32)
Ever purposely injure other		1.44 (0.62–3.35)
Other non-specified trauma**		
Ever experience other trauma	–	1.78 (0.87–3.65)
Ever trauma not want to report	–	1.81 (1.06–3.09)*

*OR, Odds Ratio; CI, Confidence Interval*.

*p < 0.05; ** p < 0.01; *** p < 0.001.

#### Ethnic Differences in PTSD Symptomatology

Table 4 displays ethnic symptom differences in Black Americans suffering from PTSD. African American and Black Caribbean PTSD sufferers' symptomatology was largely congruent, with almost no significant differences noted. The two exceptions include African Americans reporting more unpleasant dreams related to the unpleasant event and being unable to remember important parts of the traumatic event compared to Black Caribbeans. Of note, the top four most frequently reported PTSD symptoms among Black Americans were: trying not to think about what happened; having repeated unwanted memories related to the event; purposely staying away from places, people or activities that remind one of the event; and getting very upset when reminded of the past traumatic event.

**Table 4:** Sex and ethnic differences in PTSD symptomatology for Black Americans by sex and ethnicity.

	**African Americans**			**Black Caribbeans**		
	**Men**	**Women**	**Total**	**Men**	**Women**	**Total**
	**%(*N*)**	**%(*N*)**	**%(*N*)**	**%(*N*)**	**%(*N*)**	**%(*N*)**
Try not to think about (it/what happened)?^†^	62.35 (255)	70.10 (618)	66.97 (873)	65.43 (125)	71.81 (279)	68.74 (404)
Purposely stay away from places, people or activities that reminded you of (it/ the event/ this experience^†^^†^^†^	37.07 (161)	50.09 (435)	44.83 (596)	44.33 (75)	42.67 (182)	43.47 (257)
Unable to remember some important parts of what happened? *	25.06 (105)	26.99 (236)	26.21 (341)	17.45 (43)	20.15 (83)	18.85 (126)
Lose interest in doing things you used to enjoy^†^^†^^†^	29.45 (123)	42.04 (366)	36.96 (489)	34.21 (46)	34.14 (139)	34.17 (185)
Feel emotionally distant or cut-off from other people^†^^†^^†^	29.10 (121)	45.56 (393)	38.90 (514)	34.33 (44)	34.80 (146)	34.57 (190)
Have trouble feeling normal feelings like love, happiness, or warmth toward other people?^†^^†^^†^	25.38 (100)	40.74 (345)	34.53 (445)	27.38 (46)	27.87 (123)	27.63 (169)
Had no reason to plan for the future because you thought it would be cut short^†^^‡^	14.51 (62)	19.50 (167)	17.49 (229)	21.04 (25)	11.81 (53)	16.28 (78)
Ever have repeated unwanted memories of (it/ the event/ this experience/^†^^†^^†^	49.28 (153)	66.34 (489)	59.85 (642)	63.02 (67)	57.83(192)	60.21 (259)
Ever have repeated unpleasant dreams about (it/ the event/ this experience *^†^^†^	36.78 (117)	48.35 (352)	43.94 (469)	36.29 (48)	32.61 (130)	34.29 (178)
Have flashbacks – that is, suddenly act or feel as if (it/ the event/ this experience/ WORST EVENT) were happening all over again?^†^^†^^†^	42.39 (133)	54.32 (401)	49.79 (534)	53.03 (56)	50.37 (172)	51.58 (228)
Get very upset when you were reminded of (it/ the event/ this experience^†^^†^^†^	43.21 (133)	60.68 (442)	54.03 (575)	61.48 (71)	57.51 (188)	59.33 (259)
When you were reminded of (it/ the event/ this experience/ WORST EVENT), did you ever have physical reactions like sweating, your heart racing, or feeling shaky **^†^^†^^†^	25.22 (75)	38.69 (281)	33.58 (356)	21.49 (30)	30.26 (105)	26.25 (135)

*p < 0.05; **p < 0.01; ***p < 0.001 for significant ethnicity differences.

*†p < 0.05; ††p < 0.01; †††p < 0.001 for significant sex differences among African Americans*.

*‡p < 0.05; ‡‡p < 0.01; ‡‡‡p < 0.001 for significant sex differences among Black Caribbeans*.

#### Sex Differences in PTSD Symptomatology

Table 4 also displays sex differences in Black Americans suffering from PTSD. Among African Americans, there were significant differences in PTSD symptomatology between men and women, with a greater proportion of women disclosing all but one symptom–failing to remember important parts of one's traumatic experience (differences ranging between 17.5 and 5.0%). Conversely, the PTSD symptomatology frequency reported by Black Caribbean men and women held no significant differences, with one exception. Black Caribbean men were more likely to report feeling as though they had no reason to plan for the future because they felt their lives would be cut short.

#### Help-Seeking for the Worst Traumatic Event and PTSD

Overall, 45.5% of African Americans and 52.1% of Black Caribbeans obtained professional help for their worst traumatic event. Among African Americans 44.6% of men and 45.8% of women obtained professional help. Among Black Caribbeans 65.1% of men and 43.1% of women obtained professional help. None of these differences in percentages were significant.

In such cases where professional help was accessed for dealing with one's traumatic event(s), Black Americans most commonly sought assistance from psychiatrists (54.8%), mental health professionals (50.4%), family doctors (47.7%), other health professionals (17.2%), other medical doctors (17.1%), and other healers (1.7%). Family and friends (59.9%), as well as ministers (40.1%), proved other common sources of non-professional help. Of interest, African Americans were significantly more likely to seek help from a minister compared to Black Caribbeans (41.9 vs. 18.9%, respectively). But if Black Caribbeans did access a minister, women were significantly more likely than men to do so. Moreover, African American women were more likely to seek the help of an “other health professional” compared to African American men. There were no other significant ethnic or sex differences in professional help sought within the study.

Among those who did not seek help in coping with their PTSD specifically: 23.5% described thinking the problem would get better on its own; 11.7% preferred to solve the problem on their own; 9.8% did not think treatment would help; 6.1% did not know where to go for assistance; 4.7% could not afford treatment; 4.7% worried about what others would think or were embarrassed; 4.0% reported that the problem got better on its own; 2.5% noted that the problem did not bother them or was not serious enough to seek help; and 1.0% noted it was inconvenient for them to seek help.

## 4. Discussion

As a whole, several significant ethnic and sex differences in exposure to potentially traumatic events were identified within this study. African American respondents were more likely to experience spousal abuse and exposure to toxins than their Black Caribbean counterparts. Black Caribbeans reported higher lifetime exposure to muggings, natural disasters, harsh parental discipline, being a civilian living in terror and/or being a refugee than African American respondents. Specific to sex, Black men reported more events of combat, a peacekeeper/relief worker, being mugged, exposed to toxins, seeing atrocities, and/or injuring someone. Conversely, Black women were more likely to have been rape/sexual assault and/or intimate partner violence victims. The assaultive violence trauma type was most predictive of lifetime PTSD diagnosis among Black Americans.

Considering PTSD symptoms, African Americans reported more unpleasant dreams and failure to remember parts of the traumatic experience compared to Black Caribbeans. Sex-wise, African American women were more likely to report almost all PTSD symptoms compared to African American men. However, there were almost no significant differences in symptoms reported between Black Caribbean men and women (exception, men reporting no reason to plan for the future). Further, approximately half of African Americans and Black Caribbeans sought informal and formal help for their worst traumatic event, most commonly engaging family and friends, psychiatrists, and mental health professionals. African Americans were significantly more likely to engage a minister than their Black Caribbean counterparts. But African American women were more likely than men to engage another health professional, while Black Caribbean women were significantly more likely than men to engage a minister.

See subsequent sections “potential traumatic event exposure and ethnic difference”, “potential traumatic event exposure and sex differences”, “assaultive violence and lifetime PTSD diagnosis”, “African American women and PTSD symptoms”, and “Black Americans and help-seeking” for a richer explanation of select findings as well as their situation within the broader body of extant research.

### 4.1. Potential Traumatic Event Exposure and Ethnic Differences

#### African Americans, Domestic Violence

While African American and Black Caribbean respondents reported similar overall trauma exposure rates for most potentially traumatic event types, African Americans were more likely to experience domestic violence [being badly beaten by a spouse; for a greater characterization of prevalence rates and factors associated with intimate partner violence using the NSAL sample, see (60)]. In a 2020 paper, Lacey et al. (61) revisit said findings and consider help-seeking behaviour differences between African American and Black Caribbean communities. They note that cultural expectations of secrecy, the stigma associated with needing professional help, and social consequences, particularly among Black Caribbeans immigrants, may discourage victims of domestic abuse from admitting it to others outside their inner circle. Within this community, Lacey points out that many Black Caribbeans deem such information as intimate/private, only to be shared within the family network or the church, if at all. Such beliefs may explain the slightly lower rates of reporting domestic abuse by Black Caribbeans within the NSAL sample. Of course, it is also possible that Black Caribbean partners engage in less intimate partner violence than their African American counterparts.

#### Black Caribbeans, Natural Disasters, and Harsh Parental Discipline

Black Caribbeans reported greater traumatic exposure to natural disasters. Geographically, the Caribbean islands are at greater risk of hurricanes, floods, landslides, droughts, etc. (62). In addition to the physical and psychological damages caused by such events, Caribbean country economies are heavily impacted when such disasters occur (63, 64). While the literature does not indicate trauma prevalence, it is estimated that 77% of disasters experienced in the Caribbean are natural, with almost every major city within the region having experienced a natural disaster within the past 300 years (62). While the United States is not without its own costly natural disasters, the resultant death tolls remain low and the economic resources available for recovery/repair are greater than in less-developed countries (65).

Corporal punishment is a term used to characterize physically or psychologically violent disciplinary acts exhibited by an authority figure, with the intention of modifying a child's undesirable behavior [(66); e.g., spanking, hitting]. Research links harsh parental discipline in many families to: diminished psychological and cognitive functioning (67, 68); increased aggression (69, 70), higher rates of depression, substance use and suicidal ideation (71, 72); as well as lower self esteem (73) in children. The growing evidence of negative outcomes has caused many policymakers around the world to take steps to prohibit and eliminate such practices. Nevertheless, corporal punishment has received more social support in some communities, such as in Caribbean societies [see (74) for a review of corporal punishment in the Caribbean through a sociocultural lens]. In 2010, UNICEF (75) released a report summarizing findings of child discipline in randomly selected children between the ages of 2–14 from 33 countries. They found as many as 89% of children in Jamaica, and 77% in Trinidad and Tobago had experienced some sort of violent discipline (i.e., physical punishment and/or psychological aggression) within the past month. Further, 9% of children interviewed from Jamaica and 5% from Trinidad and Tobago reported “severe” physical punishment which could include: slapping of the face, head, or ears; hitting a part of the body with a hard object; being thrown or knocked down; and/or being hit by a fist or kick. In its most severe forms, this statistic also captures children who have been choked, burned/scalded on purpose, threatened with a weapon, and/or intensely beaten. Such rates are much higher than those observed within the current NSAL study but may in part be due to normative attitudes and acceptance by Black Caribbeans of physical disciplinary practices (76) or differences between Black Caribbean immigrants in the U.S. compared to those in their home countries.

### 4.2. Potential Traumatic Event Exposure and Sex Differences

#### Witnessing Violence

Both African American and Black Caribbean men were more likely than women to report witnessing atrocities, be badly beaten by someone outside the family, and purposely or accidentally injuring another. Men respondents were also more likely to report having been in combat or a peacekeeper/relief worker in times of war. These findings are supported within the literature on veterans and veteran experiences. Specifically, Freedy et al. (77) undertook a secondary analysis that considered sex differences in lifetime trauma events among Veterans Affairs primary care patients in four VA health centres in the United States. They found men veterans were more likely than women veterans to have been in combat (53.3 vs. 13.2%), and/or provided service in a war zone (51.0 vs. 9.3%). Moreover, like the current study using the NSAL data, despite the increased exposure, men and women veteran participants were equally likely to witness a severe injury or death (37.7 vs. 33.5%, respectively). Of note, approximately 16% of active American service members are Black and yet there is a lacuna of research on how Black veterans' experiences differ from that of other ethnoracial groups (78, 79).

Beyond war-related violence, the literature suggests that Black men face an increased risk of experiencing community violence, with homicide being the leading cause of death for Black men between 10 and 34 (80). While still the second leading cause of death within women of the same age range, the risk to women is much lower (41.2% of deaths in men vs. 11.7% of deaths in women). These higher homicide rates are likely to translate into an increased risk of other Black men bearing witness to an atrocity, being badly beaten and/or purposely or accidentally injuring someone. Moreover, emerging from the recent deaths of Black individuals at the hands of police officers comes research suggesting a Black person does not need to have known the victim to experience deep trauma (81). Known as vicarious traumatization, exposure to traumatic videos of police brutality online has been linked to higher levels of PTSD and depressive symptoms among Black adolescents (82). In Black adult men, a qualitative study found 95% of participants reported PTSD-related symptoms from just hearing, seeing, or reading about the fatal shooting of Stephon Clark in 2018 (83).

#### Black Women, Sexual Assault and Intimate Partner Violence

This study's results indicated similar prevalence rates between African American and Black Caribbean women on reports of rape (17.6 and 16.9%, respectively), other sexual assault (19.6 and 18.9%), or being badly beaten by a spouse (17.9 and 12.0%). In the literature, African American and Black Caribbean prevalence rates are seldom represented or differentiated.

One study that did attempt to capture the experience of intimate partner violence within African American and Black Caribbean women from health clinics in three cities: Baltimore, St. Thomas, and St. Croix (84). Authors reported an intimate partner abuse lifetime rate of 40% and an intimate partner violence lifetime rate of 27%. These event categories seem to involve broader definitions than our study (e.g., psychological abuse) which may explain the higher rates noted. Also, given the participants were recruited in a healthcare setting, and abused women are more likely to have need of healthcare services, it is likely abuse victims are overrepresented within that sample.

### 4.3. Assaultive Violence and Lifetime PTSD Diagnosis

In this sample, analyses demonstrate that assaultive violence is more likely to predict lifetime PTSD diagnosis than other forms of trauma. In the literature, this finding is supported. For instance, a UK study found assaultive violence, more than any other traumatic experience type, placed survivors at greater risk for lifetime PTSD diagnosis [34.1%; (85)]. Breslau and Peterson found respondents who had experienced assaultive violence as their earliest traumatic event were at much greater risk of developing PTSD compared to their non-assaultive counterparts [i.e., 16.9 vs. 4.9% respectively; (86)]. Another study using WHO World Mental Health Surveys sampled over 60,000 people from 24 different countries, assessing PTSD-onset persistence (87). They found forms of assaultive violence are particularly burdensome, with rape accounting for 10.2 person-years per 100 respondents (PY; calculated by multiplying the number of lifetime PTSD episodes/100 population by mean duration), other sexual assault accounting for 11.7 PY, being stalked 7.6 PY, and intimate partner sexual violence accounts for nearly 42.7% of all person-years with PTSD in the population (33.2 PY). Moreover, LeBouthillier (88) found that individuals with PTSD due to assaultive violence compared to other types had some of the highest rates of suicidal ideation and suicide attempts, with 41–52% of these having had thoughts of ending their lives.

In consideration of Black Americans, there is some comparative research on trauma event types and lifetime PTSD diagnosis risk. One study by McLaughlin and colleagues found that while Black Americans did have higher odds of lifetime PTSD than other ethnoracial groups, this difference was not significant when adjusting for trauma event type (30). Yet another study looking at African Americans and sexual violence specifically (a subtype of assaultive violence), noted a greater risk of lifetime PTSD diagnosis compared to other traumatic event types (89).

### 4.4. African American Women and PTSD Symptoms

This study found African American women were significantly more likely than their male counterparts to suffer from PTSD symptoms. Valentine and colleagues (90) worked to understand the risk of lifetime PTSD diagnosis for individuals with dual social disadvantages (i.e., is a woman and a member of a racialized group). Similar to the current study, African American women were found to have a higher lifetime PTSD prevalence compared to African American men. Of note, the authors adjusted for sociodemographic variables, mental health comorbidity, social support and potential traumatic event frequency in obtaining this finding. However, different from our findings, Valentine and team also found that once they adjusted for the number of potentially traumatic events, similar significant gender differences emerged between Black Caribbean women and men.

Absent ethnicity, there is some research considering broader sex differences in PTSD symptomatology. Generally, Guina and team found that among those with experiences of trauma, women present with more severe symptoms (91). However, after controlling for trauma type, only physical reactivity, startle response, external and internal avoidance and psychological reactivity symptoms still presented sex differences. Further, none of these sex differences were maintained in participants with formal PTSD diagnoses.

Other studies looking more granularly at sex differences in specific trauma types have maintained that sex differences exist. For instance, a study considering active-duty military personnel found women reported greater distress than their male counterparts on most PTSD symptoms (except hypervigilance) after traumatic combat-related events (92). Another study considered sex differences in PTSD symptoms following a terrorist attack (93). They found female participants reported higher levels of all PTSD symptoms, most especially re-experiencing, and anxious/dysphoric arousal. Finally, researchers followed survivors of serious motor vehicle accidents (94). They noted that aside from the re-experiencing criterion, female crash survivors were more likely to meet avoidance and arousal PTSD criteria than male crash survivors.

In consideration of why these sex differences in PTSD symptomatology exist, Olff and team suggest several hypotheses (95), including women's: tendency towards dissociation at the time of/following a traumatic event; subjective appraisals of greater threat and loss of control in response to a traumatic event; limited access to necessary social supports following a traumatic experience, and sex-specific acute psychobiological stress responses to trauma. However, while said hypotheses may explain why the current study saw increased reporting in African American women over African American men, it begs the question why similar findings were not observed between Black Caribbean women and men.

### 4.5. Black Americans and Help-Seeking

This study found near half of Black Americans sought professional assistance for their worst traumatic event. For the many that did not, most believed the problem would resolve on its own, preferred to solve the problem by themselves, or doubted treatment could help. In a recent systematic review by Smith and team (96), researchers compiled 10 principal barrier themes to help-seeking for individuals with PTSD. Of the themes noted, pride in self-reliance or perceived self-efficacy was strongly associated with a reluctance to seek professional help. Individuals believed they could “handle it on their own,” “had personal ways of coping” or that their issues were “insignificant” compared to others. Smith also cited misgivings about treatment as another barrier to help-seeking. More specifically, individuals reported feelings of hopelessness over the success of treatment, or beliefs that professional help be reserved for extreme problems only, which did not include PTSD. Moreover, others felt strongly that mental health providers would not understand their issues, not believe their trauma narratives, or opt to prescribe medications in place of considering other treatment options.

Although Smith's review did not focus exclusively on findings related to Black communities, similar reasons have been given by African Americans for failing to obtain treatment for other conditions [e.g., (97)]. Further, Motley and Banks (98) undertook a systematic review of Black American male trauma survivors specifically. They found poor physical health, a lack of trust in treatment, high levels of daily crisis, and a lack of time served as individual-level barriers to help-seeking. Moreover, income and health insurance were not significantly associated with seeking professional mental health services, but the social support of loved ones was. Finally, they found that when professional treatment was accessed, medical clinics/providers, most commonly primary care practitioners, were sought. Said findings support the current study as of professional help sought, family doctors were engaged third most frequently.

### 4.6. Implications of Findings

In light of the above findings, when treating patients of colour suffering from PTSD, *clinicians* should (1) understand that many of the evidence-based tools/interventions used to assess/treat PTSD have not been informed by/validated for use in diverse groups. If they have, it is likely not to the level of granularity so as to meet the unique needs of diverse subpopulations and/or sexes (e.g., Black communities in general vs. Black Caribbean men). (2) Clinicians should also be sure to sensitively assess client risk to trauma types most likely to be experienced based on their sex and ethnic identity. (3) When available, clinicians should employ ethnicity/culture or sex-specific interventions for treatment of PTSD. Moreover, when welcomed by the client, clinicians should consider the involvement of other supportive professionals (e.g., family doctors) and non-professionals (e.g., ministers) in the therapeutic process. (4) Clinicians should recognize when accessing external resources may be more appropriate or fill a need for the client that the clinician cannot (e.g., peer to peer support, access to necessary social services). In doing so, the clinician should familiarize themselves with local cultural and sex appropriate support services/resources available for some of the most frequently reported trauma event types. For example, culturally safe battered women shelters, Black online support groups for those who have lost a loved one, etc. Finally, (5) clinicians should proactively assess for and address any barriers or reasons for ambivalence within clients in engaging mental health supports (e.g., lack of trust in the efficacy of treatments, embarrassment, or shame at needing professional help).

*Researchers* also have an important role to play in ensuring Black communities receive the quality care they deserve. To do this, the authors recommend (1) future work be undertaken to better characterize the heterogenous experiences of traumatic events and PTSD within different Black communities, including people with roots to the Caribbean, Africa, South America, and Central America. Moreover, (2) additional work should be undertaken to capture sex and gender differences more wholly within people of colour suffering from PTSD (LGBTQ+ members included). For both (1) and (2) this consists of prevalence rates, traumatic event type exposure differences, symptom severity, intervention outcomes, help-seeking behaviours, and treatment barriers. (3) When communicating sample characteristics and study outcomes within trauma research but also more broadly, researchers should also consider “diversity within diversity” and report ethnic and sex subgroup findings. Lastly, (4) there is a need for updated statistics on the Black American experience through the administration of an updated NSAL-like national survey (99–102).

### 4.7. Limitations

While the NSAL dataset remains the most comprehensive survey of Black American mental health, it is not without limitations. Given the data is now almost two decades old, one is likely to observe some differences in reports of the potentially trauma event types experienced today compared to the early 2000's. First consider the major events to have taken place within the United States surrounding the time of NSAL's administration, most notably the attacks of September 11^th^, 2001. While our sub analysis found no major differences pre and post 9/11, within NSAL's study protocol creators made mention that “the Caribbean sample had high refusal rates, especially after 11 September (a significant proportion of the total Caribbean sample was located in the New York and New Jersey areas), due partly to fears and suspicions concerning questions about possible immigration status [(56), p. 203].” Absent these responses, one would expect higher rates of exposure to toxins, natural disaster, civilian in terror, relief worker, and seeing atrocities. Therefore, generalizability of NSAL's findings to Black Americans in the early 2000's, especially Black Caribbeans, should be carefully considered. Presently, given the COVID-19 pandemic, one would expect trauma events like experiencing a life-threatening illness, having a child present with a life-threatening illness, and having someone die unexpectedly to be higher in today's context than when the NSAL was conducted (103, 104).

Also related to NSAL's age, the PTSD criteria used to render participant diagnoses were from the older fourth edition of the DSM. The psychological community is now using the fifth edition. It is, therefore, possible different lifetime PTSD diagnoses and symptomatology results would have been obtained if the more current PTSD criteria had been employed at the time of data collection.

It is possible that given the emotional and distressing nature of disclosing one's traumatic life experiences, some participants may have opted to withhold sensitive lived experiences from their interviewer (105). This failure to disclose would have resulted in an artificially lower exposure rate to traumatic events. Consider traumatic experiences of sexual assault: it is not unusual for trauma survivors to withhold sharing said experiences with authorities, health professionals and loved ones for long periods after the traumatic event's occurrence, let alone strangers (106). Especially if the survivor believes the potential recipient of disclosure to be dissimilar to them [e.g., if the interviewer was not Black or not of the same sex; (107)]. However, on the point of interviewee vs. interviewer similarity, efforts were made by the NSAL's creators to match interviewers with participants by key social factors like race, class, and geography as a means of increasing participant comfort (56).

## 5. Conclusion

A review of the trauma literature suggests very little attention has been paid to the diverse identities present within people of colour. Much of what makes up our understanding of PTSD development, symptomatology, treatment, and outcomes is informed by research and experience working with White, cisgender, heterosexual, male participants/clients. Moreover, those that do recognize patient diversity and make strides to expand our knowledge of diverse lived experiences, often fail to consider that labels such as “non-White,” “Black,” “Asian,” and “Hispanic/Latino” mask much additional diversity within these groups. Consider the umbrella term “Native American.” Within said group there are over 570 federally recognized tribes (108). These tribes each have unique beliefs, ways of life, languages, traditions, histories, etc. that shape how they view and interact with the world. This study's findings support the presence of significant differences in subgroups when considering lifetime potential trauma event exposure between African American and Black Caribbean men and women in the United States. This study also supports the presence of some ethnic and sex differences regarding PTSD symptom presentation and help-seeking behavior. Such findings, therefore, call for clinicians and researchers to consider diversity within diversity, making targeted/tailored prevention and treatment strategies for PTSD possible for Black communities.

## Data Availability Statement

The data analyzed in this study is subject to the following licenses/restrictions: Data must be requested. Requests to access these datasets should be directed to https://www.icpsr.umich.edu/web/ICPSR/studies/20240/versions/V8.

## Ethics Statement

The studies involving human participants were reviewed and approved by University of Michigan's Institutional Review Board. The patients/participants provided their written informed consent to participate in this study.

## Author Contributions

RJT accessed the data, analysed it, and organized the output into accessible tables. SGR further analysed said data output to discover trends, led the manuscript preparation, and review of the relevant literature, with support from GJ. MTW provided manuscript guidance and direction. All authors reviewed and edited varied drafts of the manuscript.

## Funding

The data collection for this study was supported by the National Institute of Mental Health (NIMH; U01-MH57716), with supplemental support from the Office of Behavioral and Social Science Research at the National Institutes of Health (NIH) and the University of Michigan. The preparation of this article was supported by a grant from the National Institute on Aging to RTJ (P30 AG015281). This research was undertaken, in part, thanks to funding from the Canada Research Chairs Program, Canadian Institutes of Health Research (CIHR) grant number 950-232127 (MTW). Also, Canada Graduate Scholarships-Doctoral (CGS-D) Program, Social Sciences and Humanities Research Council (SSHRC) Grant Number 767-2021-2327 (SGR).

## Conflict of Interest

The authors declare that the research was conducted in the absence of any commercial or financial relationships that could be construed as a potential conflict of interest.

## Publisher's Note

All claims expressed in this article are solely those of the authors and do not necessarily represent those of their affiliated organizations, or those of the publisher, the editors and the reviewers. Any product that may be evaluated in this article, or claim that may be made by its manufacturer, is not guaranteed or endorsed by the publisher.
